# Clinical spectrum and risk factors for mortality among seawater and freshwater critically ill drowning patients: a French multicenter study

**DOI:** 10.1186/s13054-021-03792-2

**Published:** 2021-10-24

**Authors:** Florian Reizine, Agathe Delbove, Alexandre Dos Santos, Laetitia Bodenes, Pierre Bouju, Pierre Fillâtre, Aurélien Frérou, Guillaume Halley, Olivier Lesieur, Maud Jonas, Florian Berteau, Jean Morin, David Luque-Paz, Rémy Marnai, Anthony Le Meur, Cécile Aubron, Jean Reignier, Jean-Marc Tadié, Arnaud Gacouin

**Affiliations:** 1 CHU Rennes, Maladies Infectieuses Et Réanimation Médicale, 35033 Rennes, France; 2 CH Vannes, Service de Réanimation Polyvalente, 56000 Vannes, France; 3 CH La Roche Sur Yon, Service de Réanimation Polyvalente, 85191 La Roche sur Yon, France; 4 CHU Brest, Médecine Intensive Réanimation, 29200 Brest, France; 5 CH Lorient, Service de Réanimation Polyvalente, 56100 Lorient, France; 6 CH Saint Brieuc, Service de Réanimation Polyvalente, 22000 Saint-Brieuc, France; 7 CH Saint Malo, Service de Réanimation Polyvalente, 35400 Saint Malo, France; 8 CH Quimper, Service de Réanimation Polyvalente, 29000 Quimper, France; 9 CH La Rochelle, Service de Réanimation Polyvalente, 17000 La Rochelle, France; 10 CH Saint Nazaire, Service de Réanimation Polyvalente, 44600 Saint Nazaire, France; 11 CH Morlaix, Service de Réanimation Polyvalente, 29600 Morlaix, France; 12 CHU Nantes, Médecine Intensive Réanimation, 44000 Nantes, France; 13 CH Le Mans, Service de Réanimation Polyvalente, 72000 Le Mans, France; 14 CH Cholet, Service de Réanimation Polyvalente, 49300 Cholet, France

**Keywords:** Drowning, ICU, ARDS, Saltwater, Freshwater, Cardiac arrest

## Abstract

**Background:**

Drowning is a global threat and one of the leading causes of injury around the world. The impact of drowning conditions including water salinity on patients’ prognosis remains poorly explored in Intensive Care Units (ICUs) patients.

**Methods:**

We conducted a retrospective multicenter study on patients admitted to 14 ICUs in the west of France from January 2013 to January 2020. We first compared demographic and clinical characteristics at admission as well as clinical courses of these patients according to the salinity of drowning water. Then, we aimed to identify variables associated with 28-day survival using a Cox proportional hazard model.

**Results:**

Of the 270 consecutive included patients, drowning occurred in seawater in 199 patients (73.7%) and in freshwater in 71 patients (26.3%). Day-28 mortality was observed in 55 patients (20.4%). Freshwater was independently associated with 28-day mortality (Adjusted Hazard Ratio (aHR) 1.84 [95% Confidence Interval (CI) 1.03–3.29], *p* = 0.04). A higher proportion of freshwater patients presented psychiatric comorbidities (47.9 vs. 19.1%; *p* < 0.0001) and the etiology of drowning appeared more frequently to be a suicide attempt in this population (25.7 vs. 4.2%; *p* < 0.0001). The other factors independently associated with 28-day mortality were the occurrence of a drowning-related cardiac arrest (aHR 11.5 [95% CI 2.51–52.43], *p* = 0.0017), duration of cardiopulmonary resuscitation (aHR 1.05 [95% CI 1.03–1.07], *p* < 0.0001) and SOFA score at day 1 (aHR 1.2 [95% CI 1.11–1.3], *p* < 0.0001).

**Conclusions:**

In this large multicenter cohort, freshwater drowning patients had a poorer prognosis than saltwater drowning patients. Reasons for such discrepancies include differences in underlying psychiatric comorbidity, drowning circumstances and severities. Patients with initial cardiac arrest secondary to drowning remain with a very poor prognosis.

**Supplementary Information:**

The online version contains supplementary material available at 10.1186/s13054-021-03792-2.

## Introduction

Drowning is defined as the process of respiratory impairment resulting from submersion or immersion in liquid [1] and represents one of the leading causes of unintentional injuries worldwide [2]. Pathophysiology includes the loss of the normal breathing pattern leading to aspiration of water into the airways and to hypoxemia that may rapidly progress to cardiac arrest [3]. Accordingly, the cornerstones of drowning management are based on acute respiratory failure and post-resuscitation syndrome treatments that make the admission in ICU necessary in a wide proportion of such patients [4].

Reported drowning-associated mortality rates vary from 31 to 74% according to studies [1, 3–7]. Despite improvement in drowning patients’ management [1, 4], the prognosis of seriously ill drowning patients remains closely correlated with the initial occurrence of drowning-related cardiac arrest [8]. Moreover, cardiac arrest in the context of drowning is mainly of hypoxic origin that may be responsible for ischemia-induced organ damage and severe residual anoxic brain damage in survivors [9]. While studies mainly focused on the consequences of drowning, circumstances as well as the environment of the drowned person deserve to be investigated. Both characteristics of the water (including location, salinity and temperature) and the circumstances of drowning such as patients’ demographics characteristics and drowning features may influence the consequences of drowning [7].

Characteristics of the water such as salinity could have an impact on the induced biological disturbances as well as on the severity of the pulmonary lesions observed at admission [10]. Furthermore, drowning in freshwater appears to be more frequently associated with a suicide attempt, which could influence the outcome [11]. However, previous studies assessing the influence of water salinity showed controversial results. Some of them suggested a higher severity in freshwater patients without exploring admission and ICU patterns [6]. More recently, a matched cohort study reported deeper hypoxemia and a trend toward higher mortality rates without reaching statistical significance in freshwater drowning patients [10]. Conversely, when comparing drowning related out-of-hospital cardiac arrests, *Dyson et al.* suggested that seawater drowning was associated with worsen outcomes [12].

We have, therefore, conducted a multicenter retrospective cohort study on patients admitted to the ICU to assess the influence of water salinity and drowning features on short-term mortality.

## Material and methods

### Study design

We conducted a 7 years retrospective multicenter study in 14 French ICUs (3 tertiary hospitals and 11 general hospitals) in the west of France (Additional file 1: Fig. S1). All consecutive adult patients (≥ 18 years old) admitted for drowning from January 2013 to January 2020 were identified through International Classification of Diseases (ICD) coding [13]. Drowning patients were defined as patients that experienced respiratory impairment from submersion or immersion in liquid in accordance with the WHO definition [2]. The ethics committee of the French Society of Intensive Care Medicine (CE SRLF 20–03) approved the study protocol. Informed consent was not required in compliance with French legislation on observational retrospective studies of anonymized data.

### Data collection and definitions

For each included patient, a standardized form was used to collect data on demographics, medical history (including the following psychiatric comorbid conditions: depressive disorders, anxiety disorders, bipolar disorders and psychotic disorders [14]). We also collected data on the drowning episode (season of the year, type of water, suspected mechanisms and circumstances, clinical findings at the scene (Coma Glasgow Scale (CGS) score, loss of consciousness, body temperature, cardiac arrest) and on initial management (duration of cardiopulmonary resuscitation (CPR) when performed, CPR before Emergency Medical Service (EMS)). Data collected on ICU admission included clinical parameters (mean blood pressure, pulse oximetry and heart rate) and biological parameters (PaO2, PaCO2, serum sodium level, and leukocyte counts). PaO2 to FiO2 ratio in non-mechanically ventilated patients was calculated by converting O2 flow to estimated FiO2 [15]. Finally, the severity of illness and organ failures were assessed using the Simplified Acute Physiology Score II (SAPS II) [16] and the Sequential Organ Failure Assessment (SOFA) score [17]. Acute respiratory distress syndrome (ARDS) was defined in accordance with international guidelines [18]. ICU clinical course and management data were also collected including the duration of invasive mechanical ventilation (MV), neuromuscular blockers agents and catecholamine use, prone positioning, acute kidney injury defined according to KDIGO criteria [19], renal replacement therapy requirement and pneumonia occurrence defined by the presence of a radiological pulmonary infiltrate persisting for more than 24 h compatible with the diagnosis of pneumonia associated with at least 3 of the following signs: Positive microbiological respiratory samples, purulent secretions, body temperature > 38 °C without other cause and leukocytes < 4000/mm^3^ or ≥ 10,000/mm^3^. Neurological status at hospital discharge or at day 28 when patients were still hospitalized was assessed by using the Cerebral Performance Category (CPC) score [20]. Finally, hospital survival status until day 28 was recorded.

### Statistical analysis

Continuous data are reported as median [interquartile ranges (IQRs)] and categorical variables as number (%). Survival rates were established by the Kaplan–Meier method and compared by the log-rank test. For univariate analysis, patients’ characteristics were compared using Mann–Whitney test for continuous variables and the Fisher’s or the Chi-square test, when appropriate, for categorical variables. Regarding survival analysis, covariables achieving a *p* value < 0.1 in the non-adjusted analysis, with no more than 10% missing data, were entered in the adjusted analysis (Age, alcoholism, respiratory disease, drug use, presumed cardiac etiology for drowning, winter or summer seasons, cardiac arrest occurrence, CPR duration, GCS, temperature, loss of consciousness, event witnessed, resuscitation before EMS, PaCO2, invasive mechanical ventilation, SAPS2 and SOFA score). A multiple backward stepwise selection procedure eliminated those variables with an exit threshold set at *p* = 0.05. Interactions between variables were checked. To handle missing values as potential confounders, missing data were imputed using a multiple imputation with chained equations. Results are expressed by hazard ratios (HR) with their 95% confidence interval (CI). All statistical analyses were two-sided, and P values less than 0.05 were considered statistically significant. Analyses were performed using R software version 4.0.4 (https://www.rproject.org).

## Results

### General characteristics

Over the study period, 270 patients were admitted to ICU for drowning in participating ICUs of the west of France. Baseline characteristics of patients are listed in Table 1. Median inclusion number per center was 18 (IQR: 14–22). Patients were mainly male 161 (59.6%) with a median age of 68 (54–75) years and 72 patients (26.7%) had at least one psychiatric comorbidity. The presumed etiology of drowning was accidental for 151 patients (55.9%), a suicide attempt for 26 patients (10.1%). Drug or alcohol intoxication and a presumed cardiac origin were, respectively, observed in 30 and 48 patients. The overall day-28 mortality was 20.4% (55/270).

**Table 1:** Characteristics of seawater and freshwater drowning patients

	All patients n = 270	Seawater patients n = 199	Freshwater patients n = 71	*p* value
**Baseline characteristics**				
Age (years)	68 (54–75)	69 (59–77)	58 (35–69)	< 0.0001
Male sex	161 (59.6)	112 (56.3)	49 (69)	0.08
At least one psychiatric comorbidity	72 (26.7%)	38 (19.1)	34 (47.9)	< 0.0001
Obesity	38 (16.7)	27 (13.6)	11 (15.5)	0.3
Alcoholism	42 (15.6)	24 (12.1)	18 (25.3)	0.01
Cardiovascular disease	29 (10.7)	20 (10)	9 (12.7)	0.51
Respiratory disease	19 (7.0)	9 (4.5)	10 (14.1)	0.29
**Etiology** Drug or alcohol intoxication Suicide attempt Presumed cardiac Presumed neurologic Accident	30 (11.7) 26 (10.1) 48 (18.7) 15 (5.8) 151 (55.9)	18 (9.6) 8 (4.2) 43 (23) 9 (4.8) 121 (60.8)	12 (17.1) 18 (25.7) 5 (7.1) 6 (8.5) 30 (42.2)	0.11 < 0.0001 0.006 0.23 0.01
**Season** Spring Summer Autumn Winter	51 (18.9) 185 (68.5) 8 (3) 26 (9.6)	27 (13.6) 158 (79.4) 4 (2) 10 (5)	24 (33.8) 27 (38) 4 (5.6) 16 (22.5)	0.0004 < 0.0001 0.21 < 0.0001
**Scene information**				
Loss of consciousness	198 (73.3)	139 (69.8)	59 (83.1)	0.04
CGS < 13	129 (47.8)	81 (40.7)	48 (87.3)	0.0002
Event witnessed	185 (68.5)	146 (73.4)	39 (54.9)	0.006
Cardiac arrest	103 (38.1)	67 (33.7)	36 (50.7)	0.017
Resuscitation before EMS	88 (85.4)	61 (91.0)	27 (75.0)	0.03
EMS called	261 (96.7)	192 (96.5)	69 (97.8)	0.99
CPR duration	17.5 (5–30)	15 (5–30)	25 (11–37)	0.037
**Clinical and laboratory findings at ICU admission**				
Temperature (°C)	35.4 (33.5–36.6)	35.5 (34–36.6)	34.5 (31.5–36.4)	0.0096
Leukocyte count (109/L)	11.3 (8.4–16.1)	11.7 (8.8–16.1)	10.2 (7.2–15.8)	0.17
PaO2/FIO2 (mm Hg/%)	142 (93–221)	141 (93–206)	162 (95–271)	0.24
PaCO2 (mmHg)	43 (38–51)	43 (38–50)	45 (39–55)	0.044
Sodium (mmol/L)	143 (138–147)	145 (141–148)	136 (132–140)	< 0.0001
Invasive MV at day 1	105 (38.9)	62 (32.2)	43 (60.6)	< 0.0001
SAPS II at day 1	36 (26–63)	34 (26–55)	52 (28–74)	0.0026
SOFA at day 1	3 (2–8)	2 (1–6)	7 (3–12)	< 0.0001
**Clinical course and ICU management**				
Duration of mechanical ventilation (days)	3 (2–6)	4 (2–6)	2 (2–5)	0.26
ARDS	102 (37.8)	59 (29.6)	43 (60.6)	< 0.0001
**ARDS severity** Mild Moderate Severe	10 (3.7) 44 (16.2) 48 (17.8)	6 (3) 25 (12.6) 28 (14.1)	4 (5.6) 19 (26.8) 20 (28.2)	> 0.99
Neuromuscular blockers	55 (20.4)	32 (16.1)	23 (32.4)	0.0058
Prone positioning ventilation	21 (7.8)	14 (7)	7 (9.9)	0.44
Need for vasopressors	80 (29.6)	47 (23.6)	33 (46.5)	0.0005
AKI	58 (21.5)	30 (15.1)	28 (39.4)	< 0.0001
RRT use	9 (3.3)	5 (2.5)	4 (5.6)	0.25
Presumed pneumonia	107 (39.8)	75 (37.7)	32 (45.1)	0.36
ICU length of stay (days)	2 (1–4)	2 (1–4)	3 (2–7)	0.14
CPC score at hospital discharge	1 (1–2)	1 (1–1)	1 (1–5)	0.0002
Day-28 mortality	55 (20.4)	29 (14.6)	26 (36.6)	0.00015

Data are presented as median (IQR: interquartiles), n (%). P values comparing patients are tested by Mann–Whitney (continuous variables) and Chi2 or Fisher tests (categorical variables)

*Abbreviations*: CPR: Cardiopulmonary resuscitation; EMS: Emergency Medical Services; GCS: Glasgow Coma Scale; SD: Standard Deviation; MAP: Mean Arterial Pressure; PaO2: arterial oxygen tension; FiO2: Fraction of inspired Oxygen; PaCO2: Carbon dioxide tension; SAPS II: Simplified Acute Physiology Score II SOFA: Sequential Organ Failure Assessment; ICU: Intensive Care Unit. ARDS: Acute respiratory distress syndrome; RRT: Renal Replacement Therapy; AKI: Acute Kidney Injury;

### Patient characteristics according to the drowning site

**Fig. 1:**
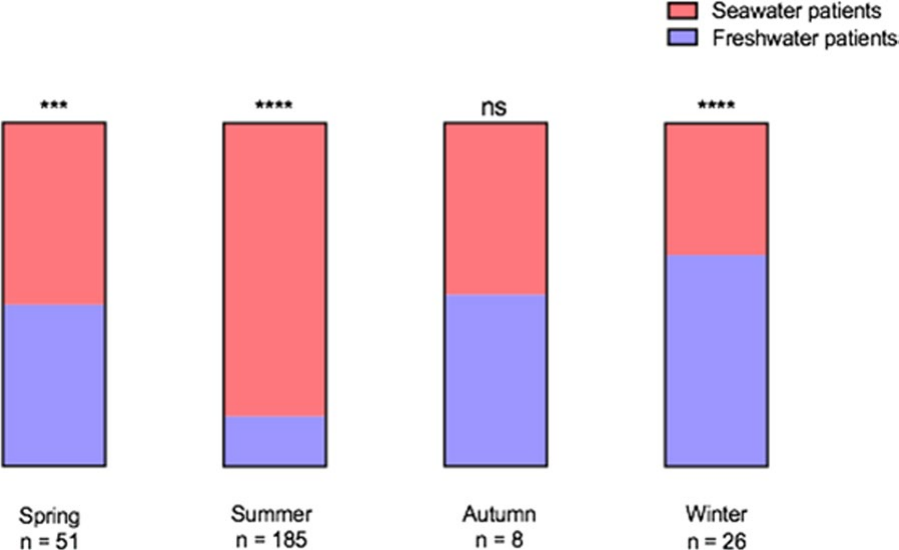
Proportion of freshwater and seawater drowning patients according to the season

Drowning occurred in seawater for 199 patients (73.7%). When comparing baseline characteristics of the patients according to the salinity of the water, freshwater drowning patients were younger and suffered more often from psychiatric comorbidities (47.9 vs. 19.1%; *p* < 0.0001). The etiology of drowning appeared more frequently to be a suicide attempt in freshwater drowning patients (4.2 vs. 25.7%; *p* < 0.0001) (Table 1). As shown in Fig. 1, seawater drowning occurred more frequently during summer (79.4 vs. 38%; *p* < 0.0001), while a higher proportion of freshwater drowning occurred during winter and spring (respectively 22.5 vs. 5%; *p* < 0.0001 and 33.8 vs. 13.6%; *p* = 0.0004).

### Severity of patients according to the drowning site

Freshwater drowning patients were more severe at the scene and in ICU than saltwater drowning patients (Table 1). They had more often an initial cardiac arrest (50.7 vs. 33.7%; *p* = 0.017), longer CPR and deeper conscious impairment at the drowning scene and, as a consequence, required more often mechanical ventilation (MV) (60.6 vs. 32.2%; *p* < 0.0001). SOFA score at ICU admission was higher in freshwater drowning patients (7 vs 2; *p* < 0.0001). A loss of consciousness was also more often observed in freshwater patients (83.1 vs. 69.8%; *p* = 0.04), and the events were less frequently witnessed in this population. When excluding patients that undergo an initial cardiac arrest, patients drowning in freshwater also appeared to be more severe (Additional 1: Table S1).

### Predictive factors for mortality at day 28

**Fig. 2:**
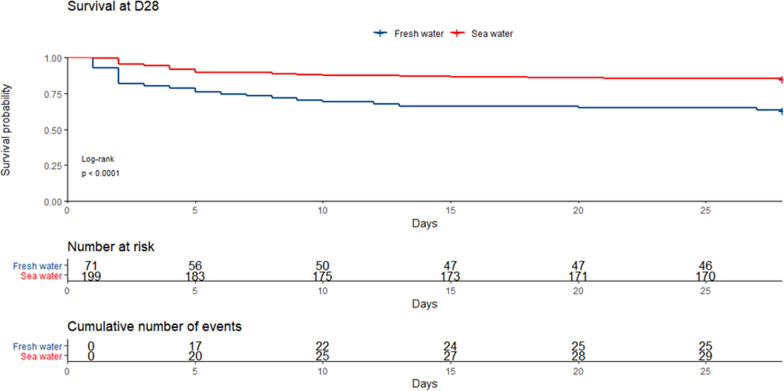
Kaplan–Meier curve reporting unadjusted influence of type of water among drowning patients

Freshwater drowning patients had worse CPC scores at hospital discharge and a higher 28-day mortality than saltwater drowning patients. Survival curves comparing seawater and freshwater drowning patients are represented in Fig. 2. Risk factors for 28-day mortality in the univariate analysis are presented in Table 2. By multivariate Cox regression, freshwater was found to be independently associated with 28-day mortality (adjusted Hazard Ratio (aHR) 1.85 [95% Confidence Interval (CI) 1.02–3.39], *p* = 0.04). The following variables were also independently associated with 28-day mortality: Occurrence of a drowning-related cardiac arrest (aHR 11.5 [95% CI 2.51–52.43], *p* = 0.0017), duration of cardiopulmonary resuscitation (aHR 1.05 [95% CI 1.03–1.07], p < 0.0001) and SOFA score at day 1 (aHR 1.2 [95% CI 1.11–1.3], *p* < 0.0001) (Table 3). Noteworthy, in freshwater drowning patients, mortality at day 28 appeared lower among patients that drowned in pools, while we observed higher mortality rates in patients that drowned in ponds (respectively, HR 0.19 [95% CI 0.06–0.64], *p* = 0.007 and HR 2.31 [95% CI 1.06–5.05], *p* = 0.03) (Additional 1: Table S2).

**Table 2:** Characteristics of drowning patients according to survival status at day-28

	All patients n = 270	Survived at Day-28 n = 215	Dead at Day-28 n = 55	HR	95% CI	*p* value
**Baseline characteristics**						
Age (years)	68 (54–75)	63.5 (58–75)	62 (48–73.5)	0.98	0.97–0.99	0.01
Male sex	161 (59.6)	123 (57.2)	38 (69.1)	1.60	0.90–2.83	0.11
At least one psychiatric comorbidity	72 (26.7%)	54 (25.1)	18 (32.7)	1.39	0.79–2.44	0.25
Obesity	38 (16.7)	29 (15.9)	9 (20)	1.12	0.44–2.83	0.8
Alcoholism	42 (15.6)	27 (12.6)	15 (27.3)	2.25	1.24–4.10	0.007
Cardiovascular disease	29 (10.7)	24 (11.2)	5 (9.1)	0.77	0.31–1.94	0.58
Respiratory disease	19 (7.0)	12 (5.6)	7 (12.7)	2.12	0.96–4.70	0.06
Epilepsy	16 (5.9)	13 (6)	3 (5.4)	0.88	0.27–2.82	0.83
**Etiology** Drug or alcohol intoxication Suicide attempt Presumed cardiac Presumed neurologic Accident	30 (11.7) 26 (10.1) 48 (18.7) 15 (5.8) 151 (55.9)	19 (9.1) 19 (9.1) 46 (22) 12 (5.7) 119 (55.3)	11 (22.9) 7 (14.6) 2 (4.2) 3 (6.2) 32 (58.2)	2.08 1.38 0.16 1.03 1.13	1.08–4.04 0.62–3.05 0.04–0.64 0.32–3.31 0.66–1.94	0.03 0.43 0.01 0.95 0.64
**Season** Spring Summer Autumn Winter	51 (18.9) 185 (68.5) 8 (3) 26 (9.6)	40 (18.6) 154 (71.3) 6 (2.8) 15 (7)	11 (20) 31 (56.4) 2 (3.6) 11 (20)	1.08 0.56 1.41 2.59	0.56–2.1 0.33–0.95 0.34–5.79 1.34–5.02	0.81 0.03 0.63 0.0047
**Scene information**						
**Type of water** Seawater Freshwater	199 (73.7) 71 (26.3)	170 (79.1) 45 (20.9)	29 (52.7) 26 (47.3)	2.93	1.72–4.97	< 0.0001
Loss of consciousness	197 (72.9)	150 (69.8)	47 (85.4)	2.97	1.35–7.5	0.01
CGS < 13	129 (47.8)	76 (35.3)	53 (96.4)	37.2	9.1–152.9	< 0.0001
Event witnessed	185 (68.5)	158 (73.5)	27 (49.1)	0.35	0.19–0.64	0.0007
Cardiac arrest	103 (38.1)	50 (23.2)	53 (96.4)	60.26	14.7–247.5	< 0.0001
Resuscitation before EMS	88 (85.4)	49 (98)	39 (70.9)	0.06	0.003–0.33	0.008
EMS called (%)	261 (96.7)	207 (96.3)	54 (98.2)	2.09	0.97–39.1	0.49
CPR duration	17.5 (5–30)	5 (2.25–10)	30 (24.5–35)	1.09	1.08–1.11	< 0.0001
**Clinical and laboratory findings at ICU admission**						
Temperature (°C)	35.4 (33.5–36.6)	35.9 (34.5–36.8)	32.4 (30.5–34)	0.69	0.64–0.75	< 0.0001
Leukocyte count (109/L)	11.3 (8.4–16.1)	11.3 (8.7–15.7)	11.1 (8–18.2)	1.00	0.99–1.01	0.82
PaO2/FIO2 (mm Hg/%)	142 (93–221)	141 (94–204)	144 (80–248)	1.00	0.99–1.00	0.47
PaCO2 (mmHg)	43 (38–51)	43 (38–49)	48 (36–61)	1.03	1.02–1.05	< 0.0001
Sodium (mmol/L)	143 (138–147)	143 (139–146)	143 (134–148)	0.99	0.95–1.03	0.51
Invasive MV at day 1	105 (38.9)	54 (25.1)	51 (92.7)	26.65	9.62–73.81	< 0.0001
SAPS II at day 1	36 (26–63)	31 (24–42)	77 (68–90)	1.07	1.06–1.09	< 0.0001
SOFA at day 1	3 (2–8)	2 (1–5)	12 (9–15)	1.37	1.3–1.45	< 0.0001

Data are presented as median (IQR: interquartiles), n (%). P values comparing patients are tested by Cox model

Abbreviations: CPR: Cardiopulmonary resuscitation; EMS: Emergency Medical Services; GCS: Glasgow Coma Scale; SD: Standard Deviation; MAP: Mean Arterial Pressure; PaO2: arterial oxygen tension; FiO2: Fraction of inspired Oxygen; PaCO2: Carbon dioxide tension; SAPS II: Simplified Acute Physiology Score II SOFA: Sequential Organ Failure Assessment

**Table 3:** Adjusted analysis for mortality at day-28

Variable	Adjusted Hazard Ratios	95% CI	*p* value
Initial cardiac arrest	11.5	2.51–52.43	0.0017
Duration of cardiopulmonary resuscitation (1-min increment)	1.05	1.03–1.07	< 0.0001
Freshwater	1.84	1.03–3.29	0.04
SOFA score at day 1 (1-point increment)	1.2	1.11–1.3	< 0.0001

## Discussion

Our large multicenter study aimed to explore demographic, clinical and biological features of 270 ICU drowning patients, the context of drowning as well as the psychiatric history of the patients seemed to have a significant impact on the prognosis of the patients.

Several studies have been performed to determine risk factors for mortality in drowning patients. These risk factors might be dichotomized as follows: those related to patient characteristics and to the course of drowning and those related to water characteristics (such as salinity, location or temperature). As reported before [8], among the main factors associated with short-term mortality, drowning-related cardiac arrest appeared to be the most important. It is well known that bystander CPR and the presence of witnesses in cardiac arrest following drowning are associated with improved neurologically favorable survival [9]. Therefore, the location of drowning may influence patients' prognosis due to the presence of trained lifeguards who can initiate early CPR whenever necessary. Of note, we found that drowning in seawater occurred more frequently during summer, the only season when lifeguards are on duty in France. Since our study was conducted in western France where swimming in freshwater is not a common recreational practice, we observed a higher proportion of intentional drowning in freshwater. Very few studies have investigated whether drowning was suicidal or not [11, 21, 22]. This important characteristic might influence the actual site of the suicide (freshwater or seawater) as well as the presence of a witness that may have an impact on the early performance of resuscitation [23]. As a consequence, we found that freshwater drowning occurred more frequently in patients with psychiatric comorbidities. Similarly, within our cohort, the proportion of patients with psychiatric comorbidities appeared to be higher among patients drowning in freshwater although this characteristic did not appear to be associated with mortality in critically ill patients [14].

Although we did not assess water temperature, when assessing the season of the year of drowning occurrence, we observed worsen outcomes among patients that drowned during cold seasons (winter). The effect of water temperature on drowning outcomes seems debated. *Quan et al.* showed better neurological outcomes among drowning patients in water > 16 °C, while in a study assessing survival at 1 month [4], *Claesson et al.* did not show any association between water temperature and survival [24].

A higher mortality rate among freshwater patients had already been described before [7, 12, 25]. Salinity of the water may affect the outcomes. First, seawater drowning associated-acute respiratory failure is mediated by the aspiration of water with a high content of sodium that may promote acute lung injury induced by alveoli inflammation, DNA damage and apoptosis [26-28]. Moreover, an experimental study assessing the severity of lung injury according to the salinity showed higher severity in seawater-drowned rabbits [29]. Almost one-third of the whole population of our patients fulfilled the criteria for ARDS [18]. However, we observed a higher proportion of patients developing ARDS in freshwater patients that could be related to the duration of immersion and the higher proportion of drowning-related cardiac arrest in these patients. Moreover, a recent review on pulmonary lesions induced by drowning highlighted the lack of evidence regarding the treatment of drowning associated ARDS [30].

Noteworthy, natremia appeared logically lower in freshwater patients, which may also have had an impact on hypoxic neurological sequelae [31, 32].

In addition to water salinity, inhalation of pathogens may also induce lung inflammation and promote the development of pneumonia. In the present study, 39.8% of patients developed a presumed pneumonia that is the most common infectious complication of drowning [33]. Some studies performed on freshwater drowning patients reported high rates of multidrug resistant microorganisms in such pneumonia [34, 35], while the largest cohort of seawater drowning associated-pneumonia showed that microorganisms found from respiratory samples are mostly bacteria with a low rate of antibiotic resistance [35]. These differences could have resulted in an inadequate empirical antibiotic therapy and worsened the outcome of freshwater drowning patients.

Some limitations have to be acknowledged. First, our study was conducted on adult ICU patients only; thus, the conclusions cannot be generalized to the whole drowning population. However, observations and results are in agreement with previous studies implying an acceptable external validity. Second, as mentioned before, our study was retrospective; this design was required due to the low incidence of the severe drowning managed in ICUs. However, the large number of participating ICUs in western France produces a reliable picture of critically ill drowning patients. Third, our analysis did not take into account the Szpilman classification, which has often been used in the past to describe the drowned [1]. However, the value of this classification in predicting the prognosis of patients other than those in cardiac arrest has recently been questioned [8]. Moreover, drowning-related cardiac arrest can be responsible for neurological sequelae leading to discontinuation of care for some patients. Practices concerning the discontinuation of care may vary according to the patients and according to the centers, which can lead to different deadlines without the origin of the death having any influence.

Finally, recent advances in the management of cardiac arrests, including preventive antibiotic use [36] and targeted temperature management [37, 38], may have improved the prognosis of cardiac arrests associated with drowning which could explain the lower mortality rate observed in this cohort than previously described [7, 10].

## Conclusion

Our large retrospective study on drowning patients managed in ICU highlights that the features of drowning as well as the salinity of drowning water have a significant impact on the fate of drowned patients. The identification of risk factors for mortality may help clinicians provide prognostic orientation.

## Supplementary Information

**Additional file 1**.** e-figure 1**. Map of participating ICUs.** e-Table 1**. Survival status at day-28 according to the location of drowning.** e-Table 2**. Baseline and hospitalization characteristics of drowning patients who did not experience initial cardiac arrest according to water salinity.

## Data Availability

The datasets from this study are available from the corresponding author on request.

## Abbreviations

aHR: Adjusted hazard ratio
AKI: Acute kidney injury
ARDS: Acute respiratory distress syndrome
CGS: Coma Glasgow Scale
CI: Confidence interval
CPC: Cerebral performance category
CPR: Cardiopulmonary resuscitation
EMS: Emergency Medical Service
HR: Hazard ratio
ICD: International Classification of Diseases
ICUs: Intensive Care Units
IQR: Interquartile ranges
MV: Mechanical ventilation
SAPS II: Simplified Acute Physiology Score II
SOFA: Sequential Organ Failure Assessment

## References

[1] Szpilman D, Morgan P (2020). Management for the Drowning Patient. Chest.

[2] Bloomberg LP, World Health Organization, editors. Global report on drowning: preventing a leading killer. Geneva, Switzerland: World Health Organization; 2014.

[3] Szpilman D, Bierens JJLM, Handley AJ, Orlowski JP (2012). Drowning. N Engl J Med.

[4] Michelet P, Bouzana F, Charmensat O, Tiger F, Durand-Gasselin J, Hraiech S (2017). Acute respiratory failure after drowning: a retrospective multicenter survey. Eur J Emerg Med.

[5] Quan L, Mack CD, Schiff MA (2014). Association of water temperature and submersion duration and drowning outcome. Resuscitation.

[6] Cerland L, Mégarbane B, Kallel H, Brouste Y, Mehdaoui H, Resiere D. Incidence and consequences of near-drowning-related pneumonia-a descriptive series from Martinique, French West Indies. Int J Environ Res Public Health. 2017;14. 10.3390/ijerph14111402 PMC5708041 29149019

[7] Quan L, Bierens JJLM, Lis R, Rowhani-Rahbar A, Morley P, Perkins GD (2016). Predicting outcome of drowning at the scene: a systematic review and meta-analyses. Resuscitation.

[8] Markarian T, Loundou A, Heyer V, Marimoutou C, Borghese L, Coulange M (2020). Drowning classification: a reappraisal of clinical presentation and prognosis for severe cases. Chest.

[9] Tobin JM, Ramos WD, Pu Y, Wernicki PG, Quan L, Rossano JW (2017). Bystander CPR is associated with improved neurologically favourable survival in cardiac arrest following drowning. Resuscitation.

[10] Michelet P, Dusart M, Boiron L, Marmin J, Mokni T, Loundou A (2019). Drowning in fresh or salt water: respective influence on respiratory function in a matched cohort study. Eur J Emerg Med.

[11] Claesson A, Krig A, Jonsson M, Ringh M, Svensson L, Forsberg S (2021). Incidence and characteristics of drowning in Sweden during a 15-year period. Resuscitation.

[12] Dyson K, Morgans A, Bray J, Matthews B, Smith K (2013). Drowning related out-of-hospital cardiac arrests: characteristics and outcomes. Resuscitation.

[13] Classification of Diseases (ICD) [Internet]. [cited 2021 Jun 15]. Available from: https://www.who.int/standards/classifications/classification-of-diseases

[14] Gacouin A, Maamar A, Fillatre P, Sylvestre E, Dolan M, Le Tulzo Y (2017). Patients with preexisting psychiatric disorders admitted to ICU: a descriptive and retrospective cohort study. Ann Intensive Care.

[15] Vincent J-L, Rello J, Marshall J, Silva E, Anzueto A, Martin CD (2009). International study of the prevalence and outcomes of infection in intensive care units. JAMA.

[16] Le Gall JR, Lemeshow S, Saulnier F (1993). A new Simplified Acute Physiology Score (SAPS II) based on a European/North American multicenter study. JAMA.

[17] Vincent JL, Moreno R, Takala J, Willatts S, De Mendonça A, Bruining H (1996). The SOFA (Sepsis-related Organ Failure Assessment) score to describe organ dysfunction/failure. On behalf of the Working Group on Sepsis-Related Problems of the European Society of Intensive Care Medicine. Intensive Care Med.

[18] ARDS Definition Task Force, Ranieri VM, Rubenfeld GD, Thompson BT, Ferguson ND, Caldwell E, et al. Acute respiratory distress syndrome: the Berlin Definition. JAMA. 2012;307:2526–33. 10.1001/jama.2012.5669 22797452

[19] Section 2: AKI Definition. Kidney Int Suppl (2011). 2012;2:19–36. 10.1038/kisup.2011.32 PMC4089595 25018918

[20] Safar P (1993). Cerebral resuscitation after cardiac arrest: research initiatives and future directions. Ann Emerg Med.

[21] Cenderadewi M, Franklin RC, Peden AE, Devine S (2019). Pattern of intentional drowning mortality: a total population retrospective cohort study in Australia, 2006–2014. BMC Public Health.

[22] Ahlm K, Saveman B-I, Björnstig U (2013). Drowning deaths in Sweden with emphasis on the presence of alcohol and drugs - a retrospective study, 1992–2009. BMC Public Health.

[23] Byard RW, Houldsworth G, James RA, Gilbert JD (2001). Characteristic features of suicidal drownings: a 20-year study. Am J Forensic Med Pathol.

[24] Claesson A, Lindqvist J, Ortenwall P, Herlitz J (2012). Characteristics of lifesaving from drowning as reported by the Swedish Fire and Rescue Services 1996–2010. Resuscitation.

[25] Bierens JJ, van der Velde EA, van Berkel M, van Zanten JJ (1990). Submersion in The Netherlands: prognostic indicators and results of resuscitation. Ann Emerg Med.

[26] Han F, Luo Y, Li Y, Liu Z, Xu D, Jin F (2012). Seawater induces apoptosis in alveolar epithelial cells via the Fas/FasL-mediated pathway. Respir Physiol Neurobiol.

[27] Li J-H, Xu M, Xie X-Y, Fan Q-X, Mu D-G, Zhang Y (2011). Tanshinone IIA suppresses lung injury and apoptosis, and modulates protein kinase B and extracellular signal-regulated protein kinase pathways in rats challenged with seawater exposure. Clin Exp Pharmacol Physiol.

[28] Liu W, Dong M, Bo L, Li C, Liu Q, Li Y (2014). Epigallocatechin-3-gallate ameliorates seawater aspiration-induced acute lung injury via regulating inflammatory cytokines and inhibiting JAK/STAT1 pathway in rats. Mediators Inflamm.

[29] Rui M, Duan Y, Wang H, Zhang X (2009). Wang Y [Differences between seawater- and freshwater-induced lung injuries]. Zhongguo Wei Zhong Bing Ji Jiu Yi Xue.

[30] Thom O, Roberts K, Devine S, Leggat PA, Franklin RC (2021). Treatment of the lung injury of drowning: a systematic review. Crit Care.

[31] Giuliani C, Peri A (2014). Effects of hyponatremia on the brain. J Clin Med.

[32] Rafat C, Flamant M, Gaudry S, Vidal-Petiot E, Ricard J-D, Dreyfuss D (2015). Hyponatremia in the intensive care unit: How to avoid a Zugzwang situation?. Ann Intensive Care.

[33] Ender PT, Dolan MJ (1997). Pneumonia associated with near-drowning. Clin Infect Dis.

[34] Tadié JM, Heming N, Serve E, Weiss N, Day N, Imbert A (2012). Drowning associated pneumonia: a descriptive cohort. Resuscitation.

[35] Robert A, Danin P-É, Quintard H, Degand N, Martis N, Doyen D (2017). Seawater drowning-associated pneumonia: a 10-year descriptive cohort in intensive care unit. Ann Intensive Care.

[36] François B, Cariou A, Clere-Jehl R, Dequin P-F, Renon-Carron F, Daix T (2019). Prevention of early ventilator-associated pneumonia after cardiac arrest. N Engl J Med.

[37] Dankiewicz J, Cronberg T, Lilja G, Jakobsen JC, Levin H, Ullén S (2021). Hypothermia versus normothermia after out-of-hospital cardiac arrest. N Engl J Med.

[38] Lascarrou J-B, Merdji H, Le Gouge A, Colin G, Grillet G, Girardie P (2019). Targeted temperature management for cardiac arrest with nonshockable rhythm. N Engl J Med.

