# Supplementary material for: Clinical spectrum and risk factors for mortality among seawater and freshwater critically ill drowning patients: a French multicenter study
Source: Crit Care. 2021 Oct 24;25:372. doi: 10.1186/s13054-021-03792-2 (PMC8543920; doi:10.1186/s13054-021-03792-2)
Supplement: Supplementary file 1 — Additional file 1. e-figure 1. Map of participating ICUs. e-Table 1. Survival status at day-28 according to the location of drowning. e-Table 2. Baseline and hospitalization characteristics of drowning patients who did not experience initial cardiac arrest according to water salinity. [file 13054_2021_3792_MOESM1_ESM.docx]

**e-figure 1. Map of participating ICUs**

**
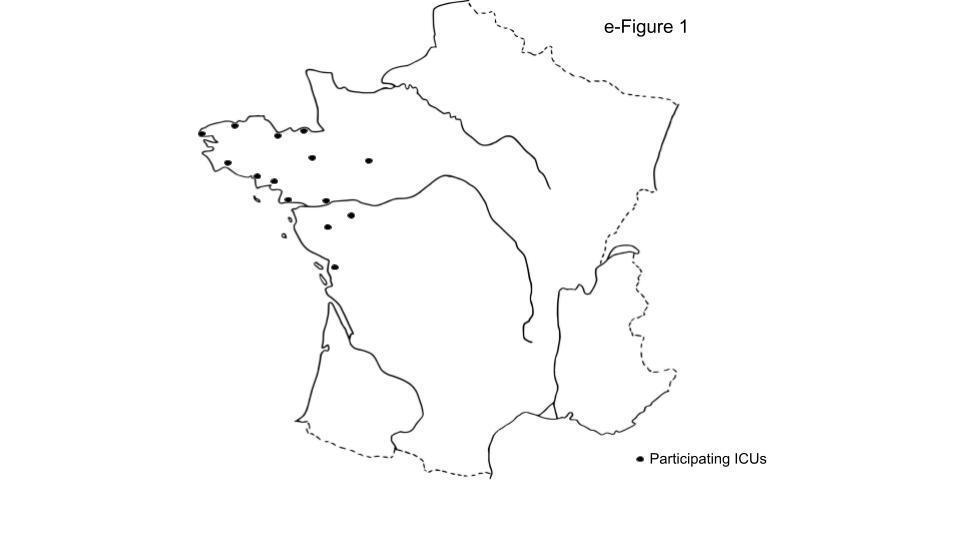
**

**e-Table 1. Survival status at day-28 according to the location of drowning**

| **Type of water** | **All patients**  **n=270** | **Survived at Day-28**  **n=215** | **Dead at Day-28**  **n=55** | **HR** | **95% CI** | ***p* value** |
| --- | --- | --- | --- | --- | --- | --- |
| **Freshwater** |  |  |  |  |  |  |
| River | 18 (25.3) | 8 (17.8) | 10 (38.5) | 2.02 | 0.91-4.46 | 0.082 |
| Pool | 25 (35.2) | 22 (48.9) | 3 (11.5) | 0.19 | 0.06-0.64 | 0.007 |
| Pond | 20 (28.2) | 9 (20) | 11 (42.3) | 2.31 | 1.06-5.05 | 0.03 |
| Other | 8 (11.2) | 6 (13.3) | 2 (7.7) | 0.62 | 0.15-2.62 | 0.62 |
| **Seawater** |  |  |  |  |  |  |
| Sea | 185 (93) | 159 (93.6) | 26 (89.7) | 0.36 | 0.21-0.61 | <0.0001 |
| Harbor | 14 (7) | 11 (6.5) | 3 (10.3) | 1.05 | 0.33-3.37 | 0.9 |

**e-Table 2. Baseline and hospitalization characteristics of drowning patients who did not experience initial cardiac arrest according to water salinity.**

|  | **All patients**  **n=167** | **Seawater patients**  **n=132** | **Freshwater patients**  **n=35** | ***p* value** |
| --- | --- | --- | --- | --- |
| **Baseline characteristics** | | | | |
| Age (years) | 69 (59-76) | 70 (65-77) | 59 (34-70) | <0.0001 |
| Male sex | 97 (58.1) | 73 (55.3) | 24 (68.6) | 0.22 |
| At least one psychiatric  comorbidity | 44 (26.3%) | 25 (18.9) | 19 (54.3) | <0.0001 |
| Obesity | 23 (13.8) | 20 (15.1) | 3 (8.6) | 0.24 |
| Alcoholism | 21 (12.6) | 14 (10.6) | 7 (20) | 0.15 |
| Cardiovascular disease | 24 (14.4) | 17 (12.9) | 7 (20) | 0.29 |
| Respiratory disease | 11 (6.6) | 6 (4.5) | 5 (14.3) | 0.054 |
| **Etiology**  Drug or alcohol intoxication  Suicide attempt  Presumed cardiac  Presumed neurologic  Accident | 12 (7.2)  17 (10.2)  35 (21)  7 (4.2)  91 (54.5) | 9 (6.8)  7 (5.3)  31 (23.5)  4 (3)  76 (57.6) | 2 (5.7)  10 (28.6)  4 (11.4)  4 (11.4)  15 (42.9) | 0.72  0.0003  0.16  0.16  0.17 |
| **Season**  Spring  Summer  Autumn  Winter | 36 (21.6)  114 (68.3)  6 (3.6)  11 (6.6) | 20 (15.1)  104 (78.8)  4 (3)  4 (3) | 16 (45.7)  10 (28.7)  2 (5.7)  7 (20) | 0.0002  <0.0001  0.61  0.0018 |
| **Scene Information** | | | | |
| Loss of consciousness | 65 (38.9) | 55 (41.7) | 10 (28.6) | 0.22 |
| GCS <13 | 33 (19.8) | 21 (15.9) | 12 (34.3) | 0.029 |
| Event witnessed | 123 (73.6) | 102 (77.3) | 21 (60) | 0.06 |
| EMS called | 158 (94.6) | 125 (94.7) | 33 (94.3) | 0.99 |
| **Clinical and laboratory findings at ICU admission** | | | | |
| Temperature (°C) | 35.9 (34.5-36.9) | 36 (34.7-37) | 35.9 (34.3-36.9) | 0.56 |
| Leukocyte count (109/L) | 11.2 (8.7-15.5) | 11.3 (9.3-15.2) | 9.9 (7.3-16.2) | 0.52 |
| PaO2/FIO2 (mm Hg/%) | 146 (94-210) | 141 (93-203) | 155 (107-251) | 0.27 |
| PaCO2 (mmHg) | 42 (38-47) | 42 (38-46) | 43 (39-50) | 0.28 |
| Sodium (mmol/L) | 142 (139-146) | 144 (141-147) | 136 (132-139) | <0.0001 |
| Invasive MV at day 1 | 33 (19.7) | 18 (13.6) | 15 (43) | 0.0002 |
| SAPS II at day 1 | 30 (24-39) | 30 (24-36) | 31 (21-52) | 0.38 |
| SOFA at day 1 | 2 (1-4) | 2 (1-3) | 5 (3-9) | <0.0001 |
| **Clinical course and ICU management** | | | | |
| Duration of mechanical ventilation (days) | 2 (1-4) | 2 (1-5) | 2 (2-4) | 0.45 |
| ARDS | 30 (18) | 18 (13.6) | 12 (34.3) | 0.011 |
| **ARDS severity**  Mild  Moderate  Severe | 4 (2.4)  15 (9)  11 (6.6) | 2 (1.5)  9 (6.8)  7 (5.3) | 2 (5.7)  6 (17.1)  4 (11.4) | >0.99 |
| Neuromuscular blockers | 10 (6) | 5 (3.8) | 5 (14.3) | 0.03 |
| Prone positioning ventilation | 4 (2.4) | 2 (1.5) | 2 (5.7) | 0.19 |
| Need for vasopressors | 19 (11.4) | 8 (6.1) | 11 (35.1) | 0.0002 |
| AKI | 14 (8.4) | 8 (6.1) | 6 (17.1) | 0.078 |
| RRT use | 1 (0.6) | 0 (0) | 1 (2.9) | 0.21 |
| Presumed pneumonia | 62 (37.1) | 45 (34.1) | 17 (48.6) | 0.17 |
| ICU length of stay (days) | 2 (1-3) | 2 (1-3) | 3 (2-5) | 0.015 |
| CPC score at hospital discharge | 1 (1-1) | 1 (1-1) | 1 (1-1) | 0.009 |
| Day-28 Mortality | 2 (1.2) | 0 (0) | 2 (5.7) | 0.04 |

Data are presented as median (IQR: interquartiles), n (%). P values comparing patients are tested by Mann-Whitney (continuous variables) and Chi2 or Fisher tests (categorical variables).

Abbreviations: CPR: Cardiopulmonary resuscitation; EMS: Emergency Medical Services; GCS: Glasgow Coma Scale; SD: Standard Deviation; MAP: Mean Arterial Pressure; PaO2: arterial oxygen tension; FiO2: Fraction of inspired Oxygen; PaCO2: Carbon dioxide tension; SAPS II: Simplified Acute Physiology Score II SOFA: Sequential Organ Failure Assessment; ICU: Intensive Care Unit. ARDS: Acute respiratory distress syndrome; RRT: Renal Replacement Therapy; AKI: Acute Kidney Injury;
